# Quantifying aluminum toxicity effects on corn phenotype using advanced imaging technologies

**DOI:** 10.1002/pld3.623

**Published:** 2024-07-22

**Authors:** Lóránt Szőke, Brigitta Tóth, Tomislav Javornik, Boris Lazarević

**Affiliations:** ^1^ Department of Plant Nutrition University of Zagreb Faculty of Agriculture Zagreb Croatia; ^2^ Institute of Food Science, Faculty of Agricultural and Food Sciences and Environmental Management University of Debrecen Debrecen Hungary; ^3^ Centre of Excellence for Biodiversity and Molecular Plant Breeding University of Zagreb Zagreb Croatia; ^4^ Department of Plant Biodiversity University of Zagreb Faculty of Agriculture Zagreb Croatia

**Keywords:** Al toxicity, chlorophyll fluorescence, corn, spectral reflectance, vegetation indices

## Abstract

Soil acidity (pH <5.5) limits agricultural production due to aluminum (Al) toxicity. The primary target of Al toxicity is the plant root. However, symptoms can be observed on the shoots. This study aims to determine the potential use of chlorophyll fluorescence imaging, multispectral imaging, and 3D multispectral scanning technology to quantify the effects of Al toxicity on corn. Corn seedlings were grown for 13 days in nutrient solutions (pH 4.0) with four Al treatments: 50, 100, 200, and 400 μM and a control (0 μM AlCl_3_ L^−1^). During the experiment, four measurements were performed: four (MT1), six (MT2), 11 (MT3), and 13 (MT4) days after the application of Al treatments. The most sensitive traits affected by Al toxicity were the reduction of plant growth and increased reflectance in the visible wavelength (affected at MT1). The reflectance of red wavelengths increased more significantly compared to near‐infrared and green wavelengths, leading to a decrease in the normalized difference vegetation index and the Green Leaf Index. The most sensitive chlorophyll fluorescence traits, effective quantum yield of PSII, and photochemical quenching coefficient were affected after prolonged Al exposure (MT3). This study demonstrates the usability of selected phenotypic traits in remote sensing studies to map Al‐toxic soils and in high‐throughput phenotyping studies to screen Al‐tolerant genotypes.

## 1 INTRODUCTION

Studies have shown that almost half of the world's potentially arable lands are acidic, and it is estimated that 50% of the world's potentially arable lands are impacted by aluminum (Al) toxicity (Panda et al., 2009; Zheng, 2010). Moreover, acidification of arable soils is a growing problem due to different agricultural practices. For example, acidification has been attributed to the long‐term use of ammonium‐nitrogen fertilizers, including urea (Tian & Niu, 2015; Zhao & Shen, 2018). Pesticides like sulfonylurea herbicides also decrease the soil pH (Grey & McCullough, 2012). Crop production also increases soil acidity because plants excessively uptake limelike elements as cations, decreasing soil pH (Daba et al., 2021).

Corn is highly susceptible to soil acidity, and Al toxicity can significantly affect corn production (Lidon & Barreiro, 2002). In acid soils, Al toxicity severely limits corn yield, often reducing it by 50%. Al toxicity in corn primarily reduces root length, subsequently decreasing water and nutrient absorption (Kochian et al., 2015; Lidon et al., 2000; Singh et al., 2017; Siqueira et al., 2020; Yang et al., 2013).

Sivaguru et al. (1999) found that Al treatment damaged plasma membranes and increased membrane permeability in corn root cells, enabling Al uptake into the symplast and impaired H^+^/NO_3_^−^ cotransport. On the cell level, Al toxicity generates reactive oxygen species (ROS) and mitochondrial dysfunction (Ranjan et al., 2021). Although the primary symptoms of Al toxicity are visible at the root level, and most studies were focused on roots (Wang et al., 2016), Al toxicity affects the whole plant's physiology.

According to Ren et al. (2022) Al toxicity affected the morphological parameters of corn by reducing the root, shoot, and total dry weight and leaf area. The lower biomass production is probably related to the impairment of the photosynthetic apparatus by Al toxicity (Ofoe et al., 2023; Panda et al., 2009). Lidon et al. (2000) further highlighted the implications of Al toxicity on nutrient accumulation in corn shoots, which can affect photosynthesis. For instance, studies have demonstrated the adverse effects of Al toxicity on photosynthetic pigments in corn. Mihailovic et al. (2008) observed a significant decrease in chlorophyll a + b (Chl‐a + b) content in Al‐sensitive corn plants under Al toxicity. Zhao et al. (2017) reported lower maximum quantum yield of primary photochemistry (F_v_/fm) and performance index of PSII in Al‐treated corn plants. Additionally, net photosynthesis showed a decline in Al‐treated corn plants (Mihailovic et al., 2008). Furthermore, Li et al. (2010) found that prolonged exposure to Al toxicity affects the photosynthetic rate of corn.

Over the last few decades, there has been a notable surge in the utilization of imaging‐based techniques to assess various abiotic stresses in crops. These techniques offer nondestructive quantification of crops' morphological, physiological, and chemical properties, enabling accurate, early detection and reliable monitoring of crop stress (Al‐Tamimi et al., 2022; Galieni et al., 2021). Also, such technology is used in high‐throughput phenotyping studies in plant breeding and understanding plant responses to abiotic stress (Araus et al., 2018; Gill et al., 2022). The increased usage of these techniques for assessing abiotic stress in crops has led to significant advancements in understanding and managing crop stress, contributing to improved crop productivity and resilience under adverse environmental conditions.

Physical and metabolic interactions within the leaf determine the reflected light's spectrum, allowing the assessment of crop stress by color and multispectral imaging (Al‐Tamimi et al., 2022).

Comparably, measures of chlorophyll fluorescence are among the most used methods for evaluating plant stress (Maxwell & Johnson, 2000), and it has been suggested that chlorophyll fluorescence is a sensitive bioindicator for determining the impacts of Al on plants (Moustaka et al., 2016). Chlorophyll fluorescence imaging has been used as a rapid, nondestructive measurement of photosynthetic performance in different plants (Al‐Tamimi et al., 2022; Chaerle et al., 2007). The combination of these techniques was previously shown as very useful in the screening for drought tolerance in Chinese silver grass (*Miscanthus sinensis* Andersson) (Lazarević, Carović‐Stanko, et al., 2022), assessment of physiological and morphological changes under drought in common bean (*Phaseolus vulgaris* L.) (Javornik et al., 2023), differentiation between drought and salinity stress in basil (*Ocimum basilicum* L.) (Lazarević et al., 2021), and classification of nutrient deficiency in common bean (Lazarević, Kontek, et al., 2022). Recently, it was shown that changes in chlorophyll content caused by Al toxicity could be remotely monitored as Al toxicity decreased normalized difference vegetation index (NDVI) in winter wheat (*Triticum aestivum* L.) (Hernández et al., 2022).

Although multispectral and chlorophyll fluorescence imaging are very powerful methods for assessing and quantifying different abiotic stresses in crops, there is limited research on using these techniques to assess plant Al toxicity.

This study aims to determine the potential use of chlorophyll fluorescence imaging, multispectral imaging, and 3D multispectral scanning technology for quantifying the effects of Al toxicity on corn. Also, the objective was to nondestructively monitor the occurrence and development of the specific Al toxicity symptoms over time and to identify Al‐sensitive traits that can be utilized for detecting Al stress.

## 2 MATERIALS AND METHODS

### 2.1 Experimental setup

The experiment was conducted in a growth chamber under controlled conditions: a 16:8 h photoperiod with a day temperature of 25°C and a night temperature of 22°C, 65% relative humidity, and 250 μmol m^−2^ s^−1^ photosynthetic photon flux density (PPFD) provided by NS12 LED lamps (Valoya Oy, Helsinki, Finland). Corn (*Zea mays*, hybrid Armagnac) seeds were germinated for 5 days between heavy‐weight seed germination papers (Anchor Paper Co., St. Paul, MN, USA). The uniformly developed plants were then chosen and transferred to a nutrient Magnavaca's nutrient solution (Magnavaca et al., 1987). Following transplantation, plants were grown 4 days in containers (70 cm long, 55 cm wide, 30 cm deep) filled with 30 L of control solution. The pH was then adjusted to 4.0 ± .1 in all containers, and aluminum (AlCl_3_) was added in varying concentrations. There were five treatments: control (0 μM), 50, 100, 200, and 400 μM AlCl_3_ L^−1^.The pH of the solutions was measured using a pH meter with the combined electrode (Mettler Toledo FE20/EL20) and adjusted each day using .1 mM hydrochloric acid (HCl) and .1 mM sodium hydroxide (NaOH), whereas solutions were replenished every 3 days. Ten uniformly developed plants were grown in each treatment and were used as replicates. All measurements were performed on each plant during four measurement times: 4 (1 MT), 6 (2 MT), 11 (3 MT), and 13 (4 MT) days after the onset of the Al treatments.

### 2.2 Chlorophyll fluorescence imaging

**FIGURE 1:**
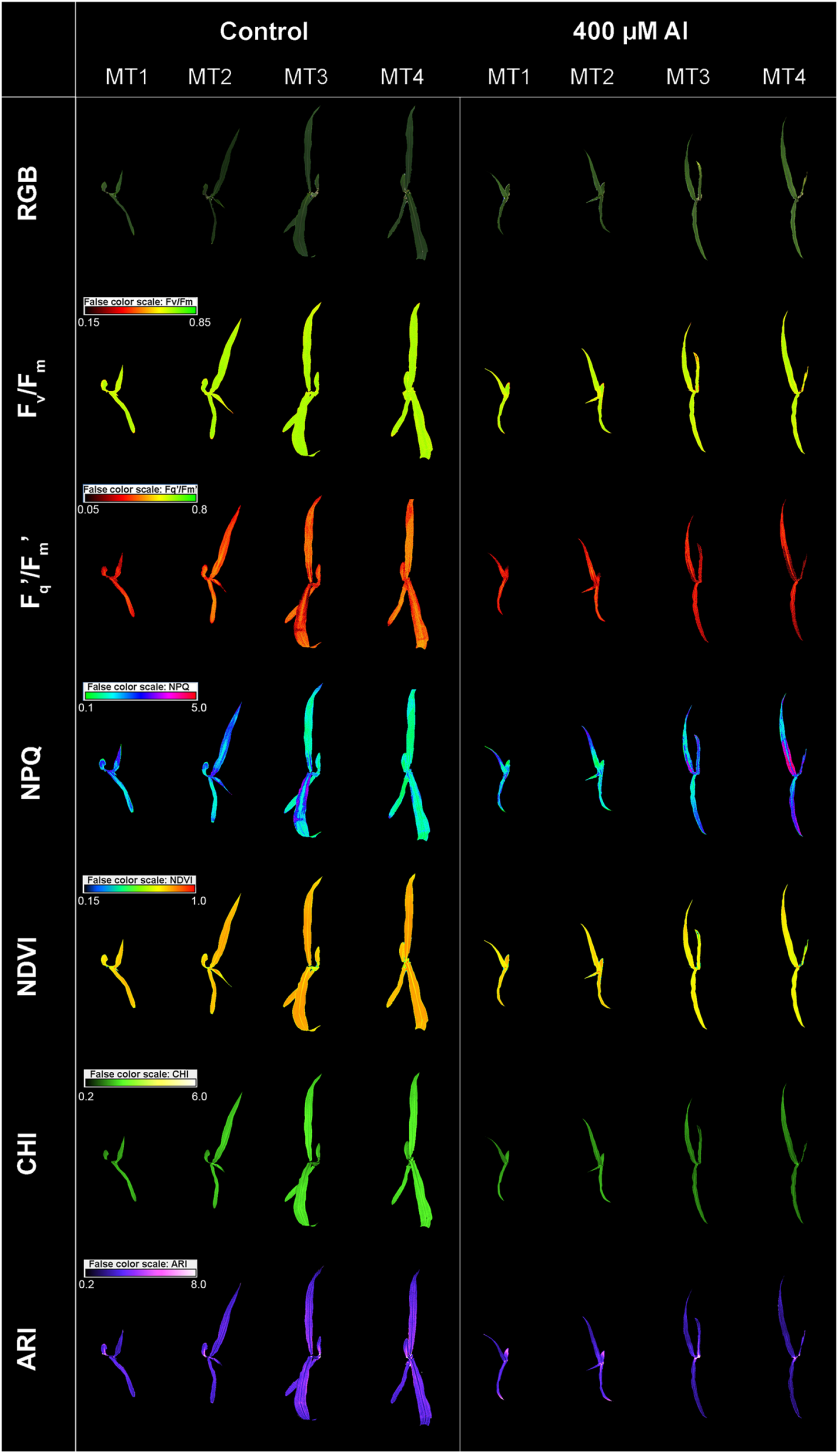
Corn color (RGB) and pseudo‐color images showing chlorophyll fluorescence parameters: the maximum quantum yield of PSII (F_v_/F_m_), the effective quantum yield of PSII (F_q_'/F_m_′), non‐photochemical quenching (NPQ), and vegetation indices: normalized difference vegetation index (NDVI); the chlorophyll index (CHI) and the anthocyanin index (ARI) captured by CropReporter™ on the fourth (MT1), sixth (MT2) 11 (MT3) and 13 day (MT4) of the experiment in control and 400 μM AlCl_3_ L^−1^ treatment.

The chlorophyll fluorescence parameters were measured by CropReporter™ (PhenoVation B.V., Wageningen, The Netherlands). All images are captured with the 10 Mp lens, 200 Lp mm^−1^ resolution, 400–1000 nm spectral range, and 1.3 Mp, 1296 × 966 pixels CCD‐camera, with real 14‐bit signal resolution. The imaging procedure was described by Lazarević et al. (2021), and it follows the protocol from Brestic and Zivcak (2013). The plants were adapted in the dark for 20 min at a room temperature of 22–24°C. Plants were imaged from 35 cm distance. For photosynthesis excitation, 4500 μmol m^−2^ s^−1^ for 800 ms of red LED light was used. To obtain the chlorophyll fluorescence image, the integration time was 200 μs. The minimum chlorophyll fluorescence (F_0_) was measured after 10 μs, and the maximum chlorophyll fluorescence (F_m_) was measured after saturation. After this, the plants were kept in the dark for 15 s and then were light‐adapted for 5 min using 250 μmol m^−2^ s^−1^ of actinic light. Before the onset of the saturating pulse, steady‐state fluorescence yield (F_s_') was measured, whereas maximal chlorophyll fluorescence (F_m_′) of light‐adapted leaves was measured at the saturation, using the saturating pulse (4500 μmol m^−2^ s^−1^). After these measurements, the light was switched off, and in the presence of far‐red light, the minimal fluorescence yield of the illuminated plant (F_0_') was estimated. From the described measurements, different chlorophyll fluorescence parameters were calculated (Table 1). Examples of imaged chlorophyll fluorescence parameters are shown in Figure 1.

**TABLE 1:** The calculated chlorophyll fluorescence parameters with abbreviations, equations, and references.

Abbreviation	Parameters	Equation
F_v_/fm	The maximum quantum yield of PSII	F_v_/fm = (F_m_ – F_0_)/fm (Kitajima & Butler, 1975)
F_q_′/fm′	The effective quantum yield of PSII	F_q_′/fm′ = (F_m_′ – F_s_')/fm′ (Genty et al., 1989)
rETR	Relative electron transport rate	rETR = F_q_′/fm′ × PPFD × (.5) (Gong & Krishnan, 2019)
NPQ	Non‐photochemical quenching	NPQ = (F_m_ – F_m_′)/fm′ (Bilger & Björkman, 1990)
qP	Coefficient of photochemical quenching	qP = (F_m_′ − F_s_')/F_v_ (Schreiber et al., 1986)
qN	Coefficient of non‐photochemical quenching	qN = 1 – (F_m_′ − F_0_′)/(F_m_ − F_0_) (Schreiber et al., 1986)
qL	Estimation of “open” reaction centers on basis of a lake model	qL = ((F_m_′ − F_s_') × F_0_')/([F_m_′ − F_0_′] × F_s_') (Kramer et al., 2004)
ɸnpq	Quantum yield of nonregulated non‐photochemical energy loss in PSII	ɸnpq = 1 − ɸpsII − 1/(NPQ + 1 + qL (Fm/Fo − 1)) (Genty et al., 1989)

Abbreviation: PPFD, photosynthetic photon flux density.

### 2.3 Color and multispectral imaging

After the chlorophyll fluorescence measurements, different spectral reflectance images were obtained by CropReporter under the actinic light (250 μmol m^−2^ s^−1^), from the same distance and using the same camera resolution. The images were captured at red (R_Red_‐640 nm), green (R_Green_‐550 nm), blue (R_Blue_‐475 nm), specific green (R_SpcGrn_‐510–590 nm), chlorophyll (R_Chl_‐730 nm), near‐infrared (R_NIR_‐769 nm), and far‐red reflectance (R_FarRed_‐710 nm). The captured multispectral images were used to calculate color and multispectral parameters and vegetation indices (Table 2). Example images of the selected multispectral parameters are shown in Figure 1.

**TABLE 2:** The measured color and multispectral parameters and calculated vegetation indices with abbreviations, equation for calculation, and the reference if appropriate.

Abbreviation	Parameter	Wavelength/equation
HUE*	Hue	HUE = 60 × [0 + (R_Green_ − R_Blue_)/(max − min)], if max = R_Red_ HUE = 60 × [2 + (R_Blue_ − R_Red_)/(max − min)], if max = R_Green_ HUE = 60 × [4 + (R_Red_ − R_Green_)/(max − min)], if max = R_Blue_ 360 was added in case of HUE <0
SAT*	Saturation	SAT = (max − min)/(max + min) if VAL >0.5, or SAT = (max − min)/(2.0 − max − min) if VAL <0.5, while max and min were selected from the R_Red_, R_Green_, R_Blue_
VAL*	Value	VAL = (max + min) /2; where max and min are selected from the R_Red_, R_Green_, R_Blue_
CHI	Chlorophyll index	CHI = (R_Chl_)^−1^ − (R_NIR_)^−1^ (Gitelson et al., 2003)
ARI	Anthocyanin index	ARI = (R_Green_)^−1^ − (R_FarRed_)^−1^ (Gitelson et al., 2001)
GLI	Green leaf index	GLI = (2 × R_Green_ − R_Red_ − R_Blue_)/(2 × R_Green_ + R_Red_ + R_Blue_) (Gobron et al., 2000)
NDVI	Normalized difference vegetation index	NDVI = (R_NIR_ − R_Red_)/(R_NIR_ + R_Red_) (Rouse et al., 1974)

Abbreviation: R_NIR_, NIR reflectance.

* Hue, saturation, and value (HSV) are components of the HSV color model, which is a way of representing colors in terms of their perceptual attributes. Hue refers to the dominant wavelength of light that produces a color. It represents the pure color without any white or black added. Saturation refers to the intensity or purity of a color. Value refers to the amount of light present in the color.

### 2.4 Morphological measurements with multispectral 3D scanning

The morphological parameters were measured by PlantEye F500 multispectral 3D scanner (Phenospex, Heerlen, The Netherlands) with the resolution: Z‐range (the distance measured from the scanner down) 40 cm, Y‐resolution (Vscan = 50 mm s^−1^) 1 mm, X‐resolution .19 mm, and Z‐resolution <1 mm. The measured parameters were plant height (mm)—calculated as the distribution of elementary triangles along the z‐axis; leaf area projected (mm^2^)—calculated as the area of the projection of all elementary triangles onto the X–Y plane; total leaf area (mm^2^)—calculated as the sum of all triangle domains, where each domain represents a group of triangles forming a unitary area; digital volume (DV) (mm^3^)—calculated as the product of the height and 3D leaf area; leaf area index (mm^2^ mm^−2^)—calculated as total leaf area/sector size; leaf inclination (LINC; mm^2^ mm^−2^) pre‐describing how erect the leaves of the plant are and calculated as total leaf area/leaf area projected; leaf angle (LANG) [degree (°)]; and light penetration depth (LPD) (mm)—measured by the deepest point at which the laser can penetrate the canopy along the z‐axis.

### 2.5 Statistical methods

The JMP® Pro 16 (SAS Institute Inc., Cary, NC, USA) software was used for statistical analysis. The analysis of variance (ANOVA) with repeated measures was done by the method of Littell et al. (2000). The model contained the fixed effects of treatment (control and Al treatments: 50, 100, 200, and 400 μM AlCl_3_) measurement time (MT1‐MT4) for repeated measurements and the interaction of treatment × measurement time. Individual plants were considered as subjects nested within treatments and modeled as random factors. The partitioned F‐tests were performed using the SLICE option to examine treatments' significance (*p* < .05) within each measurement time, followed by Tukey's HSD test.

## 3 RESULTS

Among the studied phenotypic traits, it was evident that certain characteristics exhibited a marked sensitivity or strong reaction to Al treatments. These phenotypic traits are graphically depicted within the main body of the paper. Meanwhile, the ANOVA and mean comparisons for all other measured traits are presented in Tables S1 and S2. The examples of images illustrating selected chlorophyll fluorescence traits and vegetation indices of corn grown under control and treatment with 400 μM Al during measurements (MT1‐MT4) are shown in Figure 1.

### 3.1 Al toxicity effect on the chlorophyll fluorescence parameters

**FIGURE 2:**
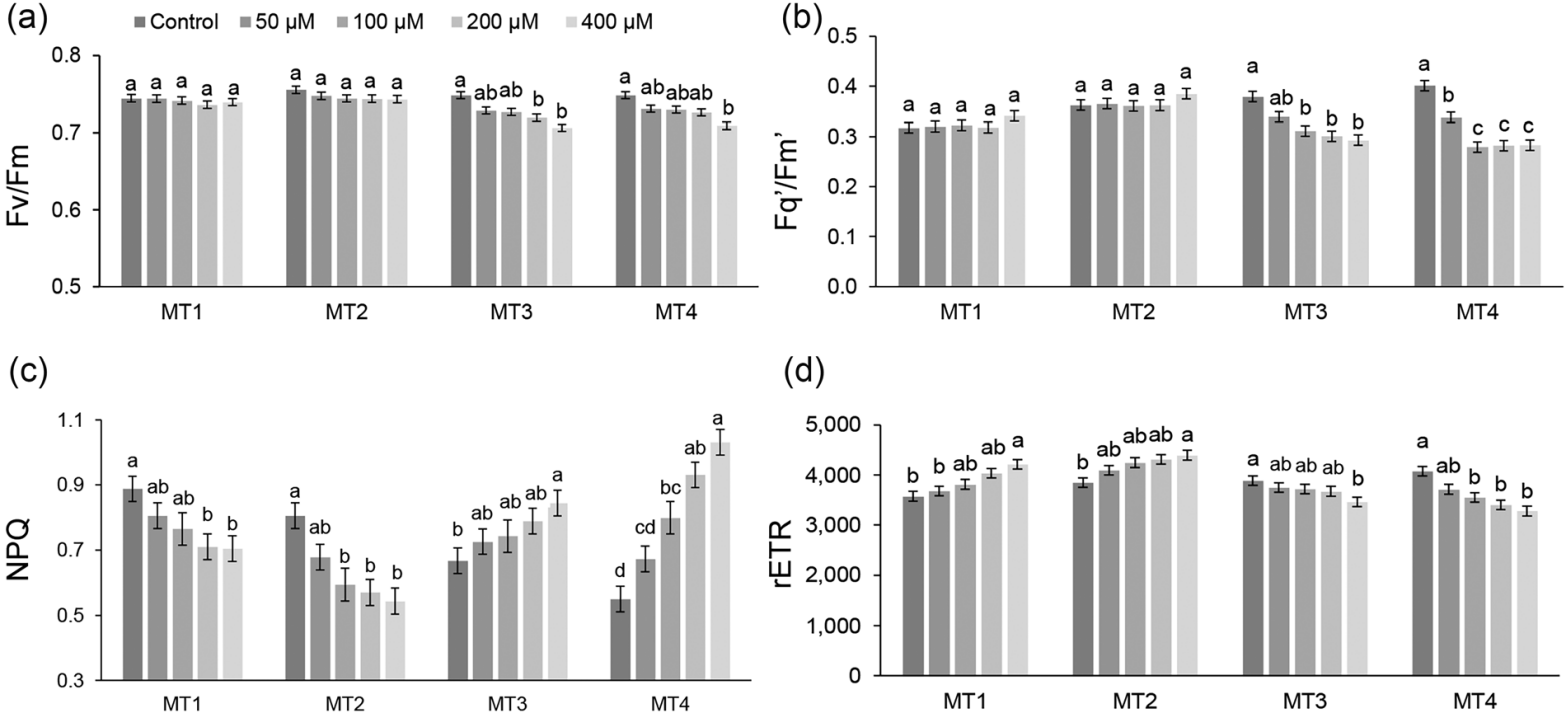
Impact of aluminum (Al) toxicity (0, 50, 100, 200, and 400 μM AlCl_3_ L^−1^) on key chlorophyll fluorescence parameters of corn ( *Zea mays* , hybrid Armagnac). Data present the means and standard errors (10 plants per treatment) for (a) maximum quantum yield of PSII (F_v_/F_m_), (b) effective quantum yield of PSII (F_q_′/F_m_′), (c) non‐photochemical quenching (NPQ), and (d) relative electron transport rate (rETR) of corn plants grown for 4 (MT1), 6 (MT2), 11 (MT3), and 13 (MT4) days in treatment solutions. Statistical significance among treatments within each measurement time is denoted by different lowercase letters (Tukey HSD test, *p* < .05).

The significant measurement time × Al treatment interaction was found for all chlorophyll fluorescence parameters except F_m_ and F_0_' (Table S1). Figure 2 displays the results for a selected chlorophyll fluorescence traits. The significant measurement time × Al treatment interaction was found mainly because Al treatments generally affected the chlorophyll fluorescence parameters after prolonged exposure (from MT3). For example, Al treatment 200 and 400 μM significantly decreased F_v_/F_m_ compared to the control at MT3 (Figure 2a). Similarly, compared to the control, a significant decrease in F_q_′/F_m_′ and qP was caused by 100, 200, and 400 μM Al treatments in MT3 and for all Al treatments at MT4 (Figure 2b and Table S2).

In first measurements (MT1 and MT2), higher Al concentrations decreased non‐photochemical quenching (NPQ, qN, and фnpq) parameters. In contrast, at MT3 and MT4, Al treatments increased non‐photochemical quenching parameters (Figure 2c; Table S2). The opposite was found for rETR, which increased with increasing Al treatments in MT1 and MT2, and after that, significantly decreased in in MT3 and MT4 (Figure 2d).

### 3.2 Al toxicity effect on the multispectral parameters and vegetation indices

**FIGURE 3:**
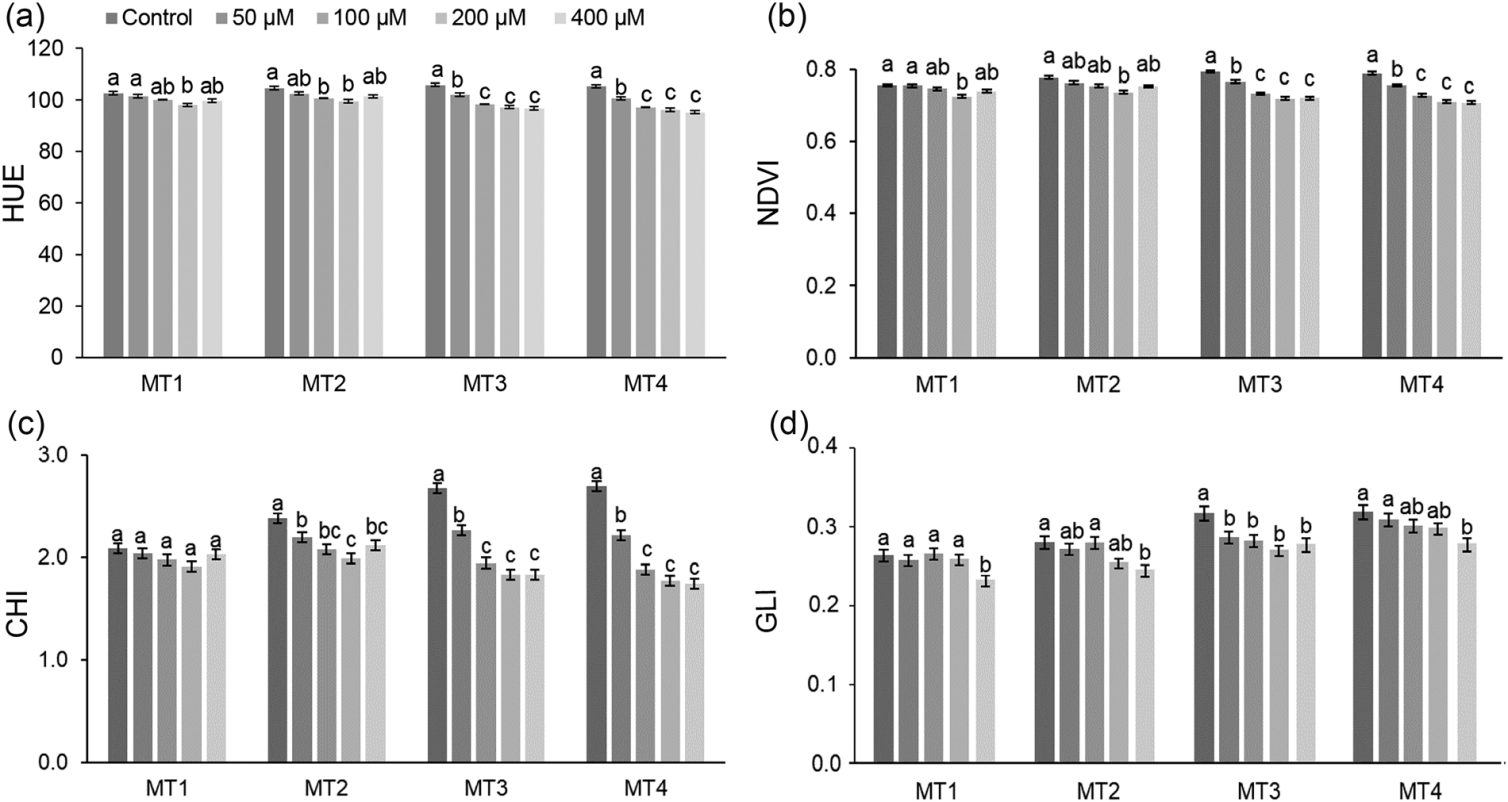
Impact of aluminum (Al) toxicity (0, 50, 100, 200, and 400 μM AlCl_3_ L^−1^) on key multispectral parameters of corn ( *Zea mays* , hybrid Armagnac). Data present the means and standard errors (10 plants per treatment) for (a) HUE (HUE), (b) normalized difference vegetation index (NDVI), (c) chlorophyll index (CHI), and (d) green leaf index (GLI) of corn plants grown for 4 (MT1), 6 (MT2), 11 (MT3), and 13 (MT4) days in treatment solutions. Statistical significance among treatments within each measurement time is denoted by different lowercase letters (Tukey HSD test, *p* < .05).

The significant measurement time × Al treatment interaction was observed for all measured multispectral parameters (Table S1). Figure 3 displays the results for HUE and selected vegetation indices (VI), and Table S2 provides the results for all assessed multispectral parameters. Al treatments generally affected all multispectral parameters and VI in all measurement times (MT1‐MT4). However, the effect was more pronounced with higher Al concentrations and later measurement times (Figure 3 and Table S2). At higher Al concentrations (200 and 400 μM) Al toxicity led to heightened spectral reflectance (R_Red_, R_Green_, R_Blue_, R_NIR_, R_FarRed_, R_SpcGrn_) (Table S2B) resulting in a notable reduction in both HUE and vegetation indices (green leaf index [GLI] and NDVI) compared to the control in MT1‐MT2 (Figure 3). These effects were even more pronounced at latter measurements (MT3 and MT4) and were also found significant at lower Al concentrations (50 and 100 μM) (Figure 3 and Table S2).

Al treatments caused significant decrease in chlorophyll content index (CHI) from MT2 (Figure 3d).

### 3.3 Effect of Al toxicity on the morphological parameters

**FIGURE 4:**
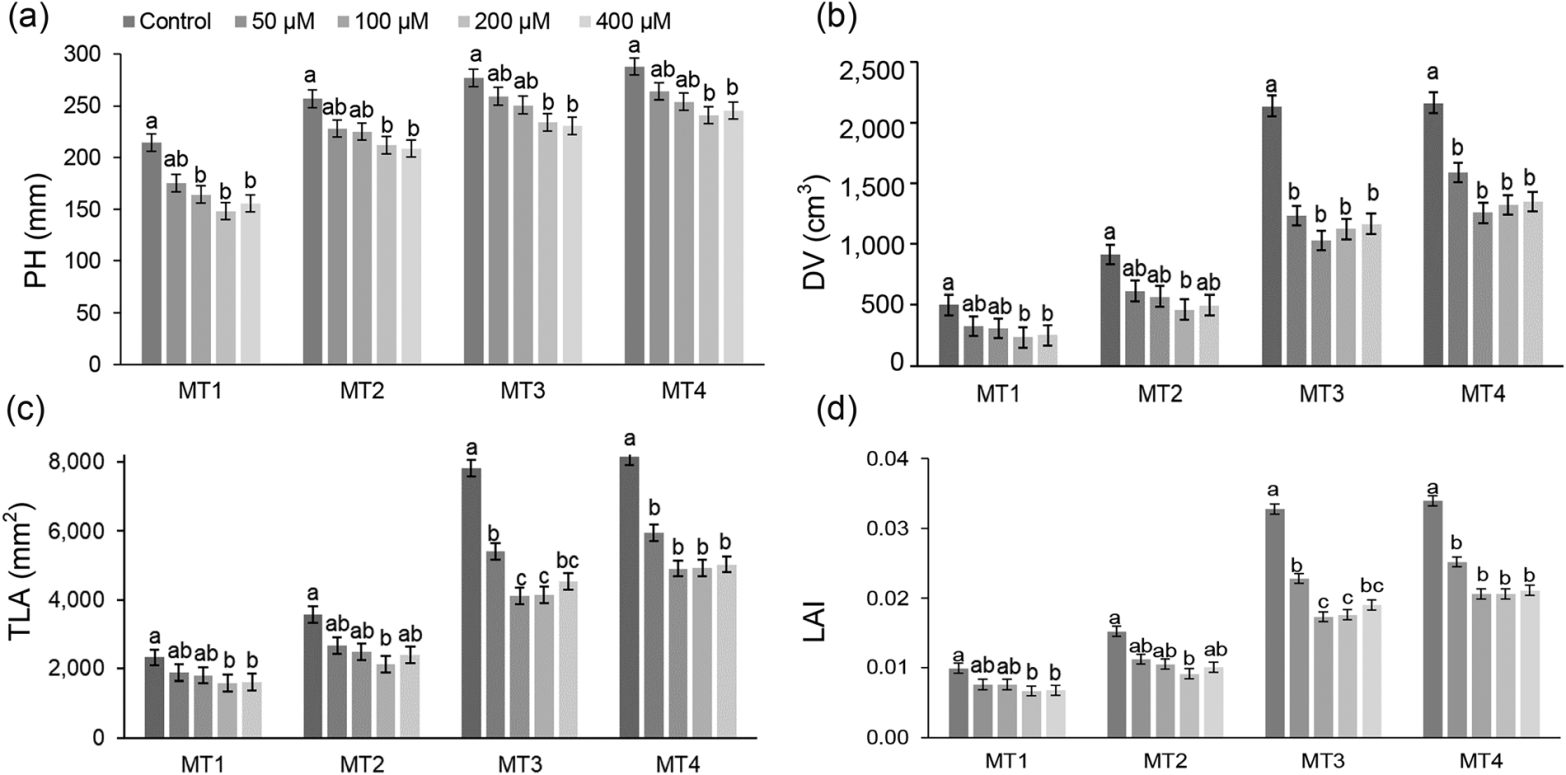
Impact of aluminum (Al) toxicity (0, 50, 100, 200, and 400 μM AlCl_3_ L^−1^) on key morphological parameters of corn ( *Zea mays* , hybrid Armagnac). Data present the means and standard errors (10 plants per treatment) for (a) plant height (PH), (b) digital volume (DV, calculated as plant height × total leaf area), (c) total leaf area (TLA), and (d) leaf area index (LAI) of corn plants grown for 4 (MT1), 6 (MT2), 11 (MT3), and 13 (MT4) days in treatment solutions. Statistical significance among treatments within each measurement time is denoted by different lowercase letters (Tukey HSD test, *p* < .05).

The significant measurement time × Al treatment interaction was found for all measured morphological parameters except for LANG and LINC (Table S1). Figure 4 displays the results for a few selected morphological parameters. The earliest effect of Al toxicity on morphological parameters was found for plant height (PH), DV, and leaf area (Figure 4), which were already reduced at MT1 in Al treatments 200 and 400 μM. From MT2, Al treatments decreased LANG and increased LINC (Table S2).

## 4 DISCUSSION

As soil acidification continues, Al toxicity is becoming more prevalent in agriculture, which will greatly impact corn productivity (Batista et al., 2013). The main site of Al toxicity in plants is the root system (Kochian et al., 2015). Thus, the primary objective of earlier research has been to gain insight into the mechanisms underlying plant tolerance and responsiveness to Al toxicity at the root level. However, root phenotyping is complex and often destructive due to its “hidden” nature beneath the soil, challenging direct observation and measurement (Atkinson et al., 2019), especially under field conditions.

Al toxicity symptoms are also presented at the shoot/leaf level due to its impairment with the photosynthetic pigments and apparatus (Ofoe et al., 2023; Panda et al., 2009). This fact enables the detection and monitoring of Al toxicity symptoms by remote sensing technology.

Compared to the root symptoms, which occurs within a few minutes or hours after root exposure to toxic Al, symptoms on aboveground organs develop over a longer period and are related to Al‐induced impairment of different metabolic processes such as nutrient and water absorption, photosynthetic capacity, and the Al‐induced oxidative stress (Ofoe et al., 2023). However, studies of the toxic effect of Al using nondestructive imaging‐based technology are scarce.

This study assessed the effect of Al toxicity on corn morphological and physiological traits using 3D multispectral scanning, multispectral imaging, and chlorophyll fluorescence imaging. Al toxicity affected almost all measured traits. Generally, the higher Al concentrations and prolonged exposure time increased the severity of the toxicity symptoms. The earliest morphological signs of Al toxicity included reduced PH, leaf area, and volume, with a corresponding decrease in LPD evident by MT1 (4 days of treatment). On the other hand, photosynthetic performance, measured by chlorophyll fluorescence parameters (F_v_/F_m_, F_q_′/F_m_′, and qP), decreased under Al treatments from MT3 (11 days of treatment).

Al toxicity in corn leads to a swift suppression of root elongation, noticeable within 30 min of exposure (Llugany et al., 1995). Such a rapid effect on shoot growth inhibition has not been reported, and the present study did not investigate the timing of the earliest occurrence in shoot growth inhibition. However, the fact that Al toxicity affected shoot growth already at MT1 (4 days of Al treatments), whereas chlorophyll fluorescence parameters were affected at prolonged exposure time, indicates that early growth reduction under Al toxicity is not related to the reduced photosynthetic process and lack of metabolic energy. Instead, it is probably caused by Al toxicity‐triggered root‐shoot signal transduction. For example, Wang et al. (2016) have shown that Al‐induced reduction of plant growth is associated with the inhibition of auxin (IAA) synthesis, transport, and imbalanced IAA distribution. Also, there are evidence for the Al stress‐induced accumulation of ABA (Salazar‐Chavarría et al., 2020), which may also suppress the plant growth under the stressful conditions (Brookbank et al., 2021). The reduction in leaf area and biomass (DV), which were observed in Al treatments from MT1, further support this and could represent a trade‐off to maintain the photosynthetic function of the leaf (Henry et al., 2019) under the Al‐induced reduction of water and nutrient absorption (Kochian et al., 2015). These early morphological trade‐offs could also explain increased rETR and decreased NPQ, qN, and фnpq at the early exposure to Al treatments (MT1 and MT2).

Reduced root, shoot, and total dry weight and leaf area were previously reported in corn under Al toxicity (Ren et al., 2022). At the same time, with the reduction of plant growth (from MT1), higher Al concentrations (200 and 400 μM L^−1^) caused an increase in R_Red_, R_Green_, R_Blue_, R_NIR_, R_FarRed_, R_SpcGrn_, and VAL.

High Al concentrations (200 and 400 μM L^−1^) decreased HUE, NDVI, and GLI from MT1 and CHI from MT2. Green leaf vegetation reflects 3% of incoming blue light, 10% of green light, and 3% of red light (Li et al., 2020), with about 90% reflecting NIR (Ustin & Jacquemoud, 2020). Lower reflectance in visible spectral bands and higher reflectance in NIR are associated with healthy chlorophyll‐rich leaves (Penuelas et al., 1995). Changes in visible light spectrum reflectance are significant, particularly for stresses damaging chlorophyll but not causing leaf yellowing (Shibayama et al., 2009).

The simultaneous increase in visible (especially R_Red_) and NIR reflectance (R_NIR_) with the decrease in NDVI indicates a substantial rise in R_Red_ compared to R_NIR_. Similarly, an increase in R_Red_, R_Green_, and R_Blue_ with a decrease in GLI indicates a higher increase in R_Red_ and R_Blue_ compared to R_Green_, suggesting a reduction in photosynthetic pigments and potential changes in leaf composition under Al toxicity. Al‐induced chlorophyll concentration reduction has been described in corn (Zahoor et al., 2017). Al inhibits aminolaevulinic acid (δ‐ALA) dehydratase, responsible for synthesizing monopyrrole porphobilinogen in chlorophyll structure, leading to reduced chlorophyll concentration in corn (Pereira et al., 2006). Both GLI and NDVI correlate strongly with corn chlorophyll content, making them useful for assessing Al toxicity.

The simultaneous increase in visible reflectance (especially R_Red_) and R_NIR_ with the decrease in NDVI indicates a substantially higher increase in reflection in the R_Red_ compared to the R_NIR_. Similarly, an increase in R_Red_, R_Green_, and R_Blue_ with the simultaneous decrease in GLI indicates a higher increase in R_Red_ and R_Blue_ compared to increase in R_Green_. All this indicates a substantial reduction in photosynthetic pigments and possible changes in structural and chemical leaf composition under Al toxicity. Al‐induced decrease in chlorophyll concentration was previously found in corn (Zahoor et al., 2017).

Mihailovic et al. (2008) found that Al toxicity caused the inhibition of aminolaevulinic acid (δ‐ALA) dehydratase, the enzyme responsible for the synthesis of monopyrrole porphobilinogen in chlorophyll structure (Pereira et al., 2006), and the concomitant reduction in chlorophyll concentration. Both GLI and NDVI are highly correlated with the corn chlorophyll content (Gitelson et al., 2014; Shaver et al., 2011), and in our study, both these indices were shown to be sensitive to Al toxicity, which makes them good candidates for the assessment of Al toxicity. Similarly, Hernández et al. (2022) used NDVI to map the Al toxicity in winter wheat in the field.

During the different measurements (from MT1 to MT4), chlorophyll fluorescence parameters NPQ, qN, фnpq, and rETR showed inconsistent behavior, namely, higher Al_3_^+^ concentrations decreased NPQ, qN, and фnpq and increased rETR in MT1 and the opposite, increased NPQ, qN, and фnpq and decreased rETR in MT3 and MT4. Similarly, the exposure to cadmium toxicity caused a decrease in NPQ, the quantum of regulated energy dissipation, and the quantum yield of nonregulated energy dissipation in two *Buddleja* species (Gong et al., 2020). These results could be explained by increased antioxidative mechanisms, such as the Mehler‐peroxidase pathway (Grace & Logan, 1996) during the early stage of the stress. This decrease in non‐photochemical quenching parameters and related electron consumption can be attributed to a reduction in the induction of NPQ, as well as a shift in the balance between the quickly and slowly reversible components of NPQ (Chen & Gallie, 2008). However, the specific sites and mechanisms of NPQ remain elusive (Nicol et al., 2019). In addition, the prolonged exposure to the Al toxicity caused an increase in NPQ and a decrease in rETR, indicating the exhaustion of these protective mechanisms. These findings suggest a complex interplay between antioxidative mechanisms and NPQ in plants, with implications for plant acclimation to Al stress.

As observed from MT3, Al toxicity has notably impacted chlorophyll fluorescence characteristics, including the maximum quantum yield of PSII (F_v_/F_m_), the effective quantum yield of PSII (F_q_′/F_m_′), and the coefficient of photochemical quenching (qP). Li et al. (2012) found that Al accumulates in chloroplasts, inhibiting electron transfer and damaging photosystem II. Zhao et al. (2017) reported similar reductions in corn's chlorophyll fluorescence and leaf photosynthesis rate. Compared to F_v_/F_m_, F_q_′/F_m_′ and the qP were found to be more sensitive to Al toxicity because a more substantial decrease was found for these parameters at lower Al concentrations. These findings are in accordance with Moustaka et al. (2016), who reported the effective quantum yield of photosystem II as the most sensitive chlorophyll fluorescence parameter to Al toxicity.

Our study indicates that Al toxicity in corn can be assessed very early (after 4 days of exposure) using nondestructive image‐based technology. However, Al toxicity in plants can cause a variety of disorders, among others: root growth inhibition, nutrient uptake and transport disruption, and oxidative stress (Bojórquez‐Quintal et al., 2017; Kochian et al., 2015; Lidon & Barreiro, 2002; Zhang et al., 2019). Therefore, the phenotypic symptoms found under conditions of Al toxicity may be caused by complex interactions between the primarily toxic effect of Al and other physiological disorders that occur under conditions of acidic soil and Al toxicity.

## 5 CONCLUSION

Given the increasing importance of acidic soils and Al toxicity in agricultural production, as well as the growing utilization of remote sensing and high‐throughput phenotyping technologies in studying complex interaction effects between crops and their environment, this study has shown that Al toxicity in corn can be assessed very early (after 4 days of exposure) using nondestructive image‐based technology. Al toxicity caused a reduction in PH, leaf area and digital plant volume, increased reflection in the visible and NIR spectrum, and decreased vegetation indices (GLI and NDVI). After prolonged exposure (11 days), Al toxicity damaged photosystem II, which has the most prominent effect on the effective quantum yield of PSII and the coefficient of photochemical quenching.

Although this study was performed under controlled conditions, and the results should be confirmed under field conditions with combinations of different stresses, such as nutrient deficiencies and oxidative stress, it demonstrated the possibility of using selected phenotypic traits in remote sensing studies to map Al‐toxic soils and/or in high‐throughput phenotyping studies to screen Al‐tolerant genotypes.

## AUTHOR CONTRIBUTIONS

*Conceptualization and methodology*: Lóránt Szőke and Boris Lazarević. *Investigation*: Lóránt Szőke, Tomislav Javornik, and Boris Lazarević. *Formal analysis*: Lóránt Szőke, Tomislav Javornik, and Boris Lazarević. *Resources*: Boris Lazarević. *Validation*: Brigitta Tóth. *Visualization*. Lóránt Szőke, Tomislav Javornik, and Boris Lazarević. *Writing original draft*: Lóránt Szőke and Boris Lazarević. *Writing, reviewing, and editing*: Brigitta Tóth, Tomislav Javornik, and Boris Lazarević. All authors approved the published manuscript. All authors have read and agreed to the published version of the manuscript.

## CONFLICT OF INTEREST STATEMENT

The authors declare no competing interests.

## Supporting information


**Data S1.** Peer Review.

**Table S1.** Analysis of variance (ANOVA) showing p‐values for measured phenotypic traits: A) Chlorophyll fluorescence traits, B) Multispectral traits, C) Morphological traits, of corn grown in aluminium treatments: control (0 μM), 50 μM, 100 μM, 200 μM and 400 μM AlCl_3_ L^−1^. Ten plants were grown in each treatment. Measurements were performed at four measurement times: 4 (MT1), 6 (MT2), 11 (MT3) and 13 (MT4) days of growth in treatment solutions.
**Table S2**. The least‐square means for partitioned F‐tests (SLICE option) to examine the significance of treatments (control (0 μM), 50 μM, 100 μM, 200 μM and 400 μM AlCl_3_ L^−1^) within measurement time (MT). For: A) Chlorophyll fluorescence traits, B) Multispectral traits, C) Morphological traits. Ten plants were grown in each treatment. Post hoc comparisons of the means were performed using Tukey's HSD test at p < .05, and different letters indicate significant difference.

## Data Availability

The data supporting this study's findings are available from the corresponding author upon reasonable request.
