# Supplementary material for: Quantifying aluminum toxicity effects on corn phenotype using advanced imaging technologies
Source: Plant Direct. 2024 Jul 22;8(7):e623. doi: 10.1002/pld3.623 (PMC11262852; doi:10.1002/pld3.623)
Supplement: Supplementary file 2 — Table S1. Analysis of variance (ANOVA) showing p‐values for measured phenotypic traits: A) Chlorophyll fluorescence traits, B) Multispectral traits, C) Morphological traits, of corn grown in aluminium treatments: control (0 μM), 50 μM, 100 μM, 200 μM and 400 μM AlCl3 L−1. Ten plants were grown in each treatment. Measurements were performed at four measurement times: 4 (MT1), 6 (MT2), 11 (MT3) and 13 (MT4) days of growth in treatment solutions. Table S2. The least‐square means for partitioned F‐tests (SLICE option) to examine the significance of treatments (control (0 μM), 50 μM, 100 μM, 200 μM and 400 μM AlCl3 L−1) within measurement time (MT). For: A) Chlorophyll fluorescence traits, B) Multispectral traits, C) Morphological traits. Ten plants were grown in each treatment. Post hoc comparisons of the means were performed using Tukey's HSD test at p < .05, and different letters indicate significant difference. [file PLD3-8-e623-s002.docx]

**Supplementary Table S1.** Analysis of variance (ANOVA) showing p-values for measured phenotypic traits: A) Chlorophyll fluorescence traits, B) Multispectral traits, C) Morphological traits, of corn grown in aluminium treatments: control (0 µM), 50 µM, 100 µM, 200 µM and 400 µM AlCl3 L-1. Ten plants were grown in each treatment. Measurements were performed at four measurement times: 4 (MT1), 6 (MT2), 11 (MT3) and 13 (MT4) days of growth in treatment solutions.

**A)**

| **Source of variation** | **Chlorophyll fluorescence traits** | | | | | | | | | | | | |
| --- | --- | --- | --- | --- | --- | --- | --- | --- | --- | --- | --- | --- | --- |
|  | **F_0_** | **F_m_** | **F_v_/F_m_** | **F_s_'** | **F_m_'** | **Fq'/Fm'** | **rETR** | **NPQ** | **F_0_'** | **qP** | **qN** | **qL** | **фnpq** |
| **Measurement Time (MT)** | <.0001 | <.0001 | <.0001 | <.0001 | <.0001 | <.0001 | <.0001 | <.0001 | <.0001 | <.0001 | <.0001 | <.0001 | <.0001 |
| **Treatment (T)** | 0.4589 | 0.0006 | <.0001 | 0.0569 | 0.0544 | 0.0002 | 0.9339 | 0.4883 | 0.0864 | <.0001 | 0.9879 | <.0001 | 0.2501 |
| **MT x T** | 0.0045 | 0.0714 | 0.0022 | 0.0020 | <.0001 | <.0001 | <.0001 | <.0001 | 0.4473 | <.0001 | <.0001 | <.0001 | <.0001 |

**B)**

| **Source of variation** | **Multispectral traits** | | | | | | | | | | | | |
| --- | --- | --- | --- | --- | --- | --- | --- | --- | --- | --- | --- | --- | --- |
|  | **R_Red_** | **R_Green_** | **R_Blue_** | **HUE** | **SAT** | **VAL** | **R_SpcGrn_** | **R_FarRed_** | **R_NIR_** | **CHI** | **ARI** | **NDVI** | **GLI** |
| **Measurement Time (MT)** | <.0001 | <.0001 | <.0001 | <.0001 | <.0001 | <.0001 | <.0001 | <.0001 | <.0001 | <.0001 | <.0001 | <.0001 | <.0001 |
| **Treatment (T)** | <.0001 | <.0001 | <.0001 | <.0001 | <.0001 | <.0001 | <.0001 | <.0001 | <.0001 | <.0001 | 0.0001 | <.0001 | <.0001 |
| **MT x T** | <.0001 | <.0001 | 0.0033 | <.0001 | <.0001 | <.0001 | <.0001 | <.0001 | <.0001 | <.0001 | <.0001 | <.0001 | 0.0006 |

**C)**

| **Source of variation** | **Morphological traits** | | | | | | | |
| --- | --- | --- | --- | --- | --- | --- | --- | --- |
|  | **DV** | **PH** | **LANG** | **LA** | **LAI** | **LAP** | **LINC** | **LPD** |
| **Measurement Time (MT)** | <.0001 | <.0001 | <.0001 | <.0001 | <.0001 | <.0001 | <.0001 | <.0001 |
| **Treatment (T)** | <.0001 | 0.0013 | <.0001 | <.0001 | <.0001 | <.0001 | 0.0004 | 0.1859 |
| **MT x T** | <.0001 | 0.0003 | 0.5957 | <.0001 | <.0001 | <.0001 | 0.8693 | <.0001 |

**Supplementary Table S2.** The least-square means for partitioned F-tests (SLICE option) to examine the significance of treatments (control (0 µM), 50 µM, 100 µM, 200 µM and 400 µM AlCl3 L-1) within measurement time (MT). For: A) Chlorophyll fluorescence traits, B) Multispectral traits, C) Morphological traits. Post hoc comparisons of the means were performed using Tukey’s HSD test at p < 0.05, and different letters indicate significant differences.

**A) Chlorophyll fluorescence traits**

| **Treatment** | **Measurement  time** | **F_0_** | | **F_m_** | | **F_s_'** | | **F_m_'** | | **F_0_'** | | **qP** | | **qN** | | **qL** | | **фnpq** | |
| --- | --- | --- | --- | --- | --- | --- | --- | --- | --- | --- | --- | --- | --- | --- | --- | --- | --- | --- | --- |
| Control | 1 MT | 2776 | A | 10946 | A | 4049 | A | 5978 | A | 2271 | A | 0.51 | A | 0.54 | A | 0.31 | A | 0.31 | A |
| 50 µM | 1 MT | 2711 | A | 10696 | A | 4190 | A | 6158 | A | 2264 | A | 0.51 | A | 0.51 | AB | 0.30 | A | 0.29 | AB |
| 100 µM | 1 MT | 2658 | A | 10362 | A | 4084 | A | 6052 | A | 2231 | A | 0.51 | A | 0.50 | AB | 0.30 | A | 0.29 | AB |
| 200 µM | 1 MT | 2724 | A | 10488 | A | 4292 | A | 6433 | A | 2307 | A | 0.50 | A | 0.48 | B | 0.29 | A | 0.27 | B |
| 400 µM | 1 MT | 2647 | A | 10338 | A | 4127 | A | 6313 | A | 2256 | A | 0.53 | A | 0.47 | B | 0.32 | A | 0.27 | B |
| Control | 2 MT | 2501 | A | 10279 | A | 3716 | B | 5924 | B | 2099 | A | 0.56 | A | 0.51 | A | 0.33 | A | 0.28 | A |
| 50 µM | 2 MT | 2468 | A | 10100 | A | 3867 | AB | 6170 | AB | 2117 | A | 0.56 | A | 0.46 | AB | 0.32 | A | 0.25 | AB |
| 100 µM | 2 MT | 2489 | A | 9854 | A | 4048 | A | 6421 | A | 2167 | A | 0.55 | A | 0.42 | B | 0.31 | A | 0.23 | AB |
| 200 µM | 2 MT | 2409 | A | 9658 | A | 3950 | AB | 6310 | AB | 2107 | A | 0.55 | A | 0.41 | B | 0.31 | A | 0.23 | B |
| 400 µM | 2 MT | 2344 | A | 9558 | A | 3847 | AB | 6355 | AB | 2068 | A | 0.57 | A | 0.39 | B | 0.33 | A | 0.21 | B |
| Control | 3 MT | 2431 | A | 9826 | A | 3685 | A | 6106 | A | 2086 | A | 0.58 | A | 0.45 | B | 0.34 | A | 0.24 | B |
| 50 µM | 3 MT | 2588 | A | 9716 | A | 3736 | A | 5784 | AB | 2161 | A | 0.54 | AB | 0.49 | A | 0.33 | AB | 0.27 | AB |
| 100 µM | 3 MT | 2642 | A | 9263 | A | 3780 | A | 5466 | B | 2162 | A | 0.51 | B | 0.49 | A | 0.30 | B | 0.29 | A |
| 200 µM | 3 MT | 2600 | A | 9661 | A | 3777 | A | 5530 | B | 2129 | A | 0.49 | B | 0.50 | A | 0.29 | B | 0.30 | A |
| 400 µM | 3 MT | 2587 | A | 9672 | A | 3650 | A | 5457 | B | 2088 | A | 0.48 | B | 0.51 | A | 0.29 | B | 0.30 | A |
| Control | 4 MT | 2327 | B | 9444 | A | 3668 | A | 6273 | A | 2052 | AB | 0.60 | A | 0.41 | D | 0.35 | A | 0.21 | C |
| 50 µM | 4 MT | 2440 | AB | 9328 | A | 3671 | A | 5710 | B | 2066 | A | 0.53 | B | 0.47 | CD | 0.31 | B | 0.26 | B |
| 100 µM | 4 MT | 2500 | AB | 9034 | A | 3656 | A | 5195 | C | 2051 | AB | 0.47 | BC | 0.50 | BC | 0.29 | BC | 0.32 | A |
| 200 µM | 4 MT | 2515 | A | 9401 | A | 3541 | AB | 5041 | C | 2007 | AB | 0.46 | BC | 0.55 | AB | 0.28 | BC | 0.34 | A |
| 400 µM | 4 MT | 2500 | AB | 9378 | A | 3394 | B | 4862 | C | 1949 | B | 0.45 | C | 0.57 | A | 0.27 | C | 0.35 | A |

Post hoc comparisons of the means were performed using Tukey’s HSD test at p < 0.05. Different letters indicate significant differences.

**B) Multispectral traits**

| **Treatment** | **Measurement time** | **R_Red_** | | **R_Green_** | | **R_Blue_** | | **SAT** | | **VAL** | | **R_SpcGrn_** | | **R_FarRed_** | | **R_NIR_** | | **ARI** | |
| --- | --- | --- | --- | --- | --- | --- | --- | --- | --- | --- | --- | --- | --- | --- | --- | --- | --- | --- | --- |
| Control | 1 MT | 2918 | C | 4703 | C | 2181 | B | 0.54 | A | 0.072 | C | 5169 | B | 6931 | B | 20964 | B | 1.12 | A |
| 50 µM | 1 MT | 3031 | C | 4780 | BC | 2220 | B | 0.54 | A | 0.073 | BC | 5288 | B | 7299 | AB | 21668 | B | 1.23 | A |
| 100 µM | 1 MT | 3171 | BC | 4909 | BC | 2271 | B | 0.54 | A | 0.075 | BC | 5399 | B | 7486 | A | 21766 | B | 1.19 | A |
| 200 µM | 1 MT | 3662 | A | 5534 | A | 2539 | A | 0.55 | A | 0.085 | A | 5866 | A | 7723 | A | 22936 | A | 1.11 | A |
| 400 µM | 1 MT | 3473 | AB | 5235 | AB | 2532 | A | 0.52 | A | 0.080 | AB | 5514 | AB | 7669 | A | 23127 | A | 1.26 | A |
| Control | 2 MT | 2622 | C | 4280 | C | 2033 | C | 0.53 | A | 0.065 | C | 4769 | C | 6265 | C | 20808 | C | 1.11 | A |
| 50 µM | 2 MT | 2882 | BC | 4578 | BC | 2176 | BC | 0.53 | A | 0.070 | BC | 5066 | BC | 6810 | B | 21305 | BC | 1.16 | A |
| 100 µM | 2 MT | 3069 | AB | 4808 | AB | 2246 | AB | 0.54 | A | 0.074 | AB | 5313 | AB | 7222 | AB | 21832 | AB | 1.17 | A |
| 200 µM | 2 MT | 3290 | A | 5017 | A | 2370 | A | 0.53 | A | 0.077 | A | 5504 | A | 7415 | B | 21644 | A | 1.11 | A |
| 400 µM | 2 MT | 3052 | AB | 4719 | AB | 2290 | AB | 0.52 | A | 0.072 | AB | 5207 | AB | 7099 | AB | 21642 | AB | 1.20 | A |
| Control | 3 MT | 2404 | C | 3893 | C | 1924 | C | 0.51 | B | 0.059 | C | 4363 | C | 5763 | C | 20870 | B | 1.22 | A |
| 50 µM | 3 MT | 2825 | B | 4504 | B | 2068 | B | 0.54 | A | 0.069 | B | 5021 | B | 6635 | B | 21210 | AB | 1.11 | AB |
| 100 µM | 3 MT | 3277 | A | 5063 | A | 2252 | A | 0.55 | A | 0.077 | A | 5593 | A | 7328 | A | 21235 | AB | 0.97 | B |
| 200 µM | 3 MT | 3521 | A | 5385 | A | 2365 | A | 0.56 | A | 0.082 | A | 5914 | A | 7750 | A | 21548 | A | 0.92 | B |
| 400 µM | 3 MT | 3471 | A | 5258 | A | 2328 | A | 0.55 | A | 0.081 | A | 5787 | A | 7702 | A | 21367 | AB | 1.00 | B |
| Control | 4 MT | 2459 | C | 3914 | C | 1958 | B | 0.50 | C | 0.060 | C | 4361 | C | 5717 | C | 20796 | A | 1.19 | A |
| 50 µM | 4 MT | 2919 | B | 4573 | B | 2074 | B | 0.54 | B | 0.070 | B | 5080 | B | 6679 | B | 20950 | A | 1.06 | AB |
| 100 µM | 4 MT | 3386 | A | 5189 | A | 2260 | A | 0.56 | AB | 0.079 | A | 5721 | A | 7430 | A | 21024 | A | 0.90 | C |
| 200 µM | 4 MT | 3591 | A | 5473 | A | 2345 | A | 0.57 | A | 0.084 | A | 6017 | A | 7780 | A | 21192 | A | 0.85 | C |
| 400 µM | 4 MT | 3599 | A | 5409 | A | 2324 | A | 0.57 | A | 0.083 | A | 5950 | A | 7836 | A | 21010 | A | 0.93 | BC |

Post hoc comparisons of the means were performed using Tukey’s HSD test at p < 0.05. Different letters indicate significant differences.

**C) Morphological traits**

| **Treatment** | **Measurement  time** | **LANG  (°)** | | **LAP  (mm^2^)** | | **LINC  (mm^2^/mm^2^)** | | **LPD  (mm)** | |
| --- | --- | --- | --- | --- | --- | --- | --- | --- | --- |
| Control | 1 MT | 38.42 | A | 1452 | A | 1.62 | A | 138 | A |
| 50 µM | 1 MT | 38.33 | A | 1141 | AB | 1.62 | A | 109 | B |
| 100 µM | 1 MT | 35.35 | A | 1047 | B | 1.74 | A | 99 | B |
| 200 µM | 1 MT | 34.38 | A | 894 | B | 1.78 | A | 86 | B |
| 400 µM | 1 MT | 35.37 | A | 961 | B | 1.77 | A | 95 | B |
| Control | 2 MT | 38.17 | A | 2224 | A | 1.63 | B | 158 | A |
| 50 µM | 2 MT | 37.49 | A | 1616 | AB | 1.65 | B | 144 | A |
| 100 µM | 2 MT | 35.38 | AB | 1440 | AB | 1.73 | AB | 144 | A |
| 200 µM | 2 MT | 34.09 | B | 1191 | B | 1.80 | A | 134 | AB |
| 400 µM | 2 MT | 34.36 | B | 1350 | AB | 1.79 | AB | 125 | B |
| Control | 3 MT | 41.41 | A | 5188 | A | 1.52 | C | 128 | B |
| 50 µM | 3 MT | 39.95 | AB | 3524 | B | 1.56 | BC | 151 | A |
| 100 µM | 3 MT | 39.76 | AB | 2624 | C | 1.57 | ABC | 159 | A |
| 200 µM | 3 MT | 36.19 | B | 2451 | C | 1.70 | A | 155 | A |
| 400 µM | 3 MT | 37.33 | B | 2755 | C | 1.66 | AB | 138 | AB |
| Control | 4 MT | 44.09 | A | 5685 | A | 1.44 | B | 126 | B |
| 50 µM | 4 MT | 40.99 | AB | 3916 | B | 1.53 | AB | 151 | A |
| 100 µM | 4 MT | 40.64 | AB | 3197 | B | 1.55 | AB | 143 | A |
| 200 µM | 4 MT | 39.53 | B | 3142 | B | 1.58 | A | 146 | A |
| 400 µM | 4 MT | 38.04 | B | 3092 | B | 1.64 | A | 148 | A |

Post hoc comparisons of the means were performed using Tukey’s HSD test at p < 0.05. Different letters indicate significant differences.
