# Supplementary material for: Quantifying aluminum toxicity effects on corn phenotype using advanced imaging technologies
Source: Plant Direct. 2024 Jul 22;8(7):e623. doi: 10.1002/pld3.623 (PMC11262852; doi:10.1002/pld3.623)
Supplement: Supplementary file 1 — Data S1. Peer Review. [file PLD3-8-e623-s001.pdf]

Plant Direct
Open Access

Quantifying Aluminium Toxicity Effects on Corn Phenotype Using Advanced Imaging Technologies

Lóránt Szőke[1,2], Brigitta Tóth[2], Tomislav Javornik[3,4], Boris Lazarević[1,3*]

Decision Letter Round 1:

June 13, 2024

Dr. Boris Lazarevic
University of Zagreb Faculty of Agriculture
Department of Plant Nutrrition
Svetosimunska cesta 25
Zagreb, N/A
Croatia

RE: Quantifying Aluminium Toxicity Effects on Corn Phenotype Using Advanced Imaging Technologies

Dear Dr. Lazarevic:

Thank you for submitting to Plant Direct. All required reviews have been returned and we have now finished our evaluation of your manuscript. In light of the reviewers' and editor's comments, further revisions are needed before the paper can be accepted for publication in Plant Direct.

Please view the editors' and reviewers' comments below and use their suggestions as a guide while you work on your revision.

When uploading the revised version of this article, please be sure to include the following:

-A word document that contains your response to the reviewers. You should respond to each reviewer comment and note the changes made to the manuscript. If you do not agree with a reviewer's comment and choose not to make a suggested revision, please explain why. Please try to provide as complete an answer as possible to each reviewer's criticisms in the "response to reviewers" section.
-A tracked changes document
- A clean version of the latest version of the manuscript

Comments from Academic Editor:
After careful review, we feel that the submitted manuscript does not fully meet Plant Direct's publication criteria. We invite you to submit a revised manuscript that addresses the points outlined by the reviewers and the academic editor.

These points include:
1. Provide appropriate details in the methods and figure legends, as appropriate, regarding both biological and technical replication (See Reviewer 1 comments).
2. Expand the Discussion to cover the potentially unexpected observation that NPQ decreases with increasing AI concentration at the first two time points (MT1 and MT2), but these parameters both decrease at increasing Al concentration at MT3 and MT4. rETR shows a similar change in Al concentration response - in this case, increasing at MT1 and MT2, and decreasing with increasing Al concentration in MT3 and MT4. Although these opposite trends for both traits were reported in the Results they are not discussed or put in context of what is reported in the literature. See Reviewer 1 comments.
3. The authors conclude that these above-ground Al-induced phenotypes identified by high-throughput imaging phenotyping could be used "in remote sensing studies to map Al-toxic soils". However, many of the measured phenotypes are likely to result from other stresses as well. It seems that additional work would be needed to identify Al-specific parameters, and some discussion on this may be needed.

Additional Minor Edits:
Line 194: HDS should be HSD (Honestly Significant Difference)
Line 248: Cite Table S2B for Rred, Rgreen, etc.
Line 268: provide a conceptual definition of "digital volume", as this is not a commonly reported parameter.
Supplemental Tables 1 and 2: State in the legend or in table what values are being presented, presumably p-values.

To upload your revision, please click the link below.
https://plantdirect.msubmit.net/cgi-bin/main.plex?el=A4Lr2hP7A5ojo2I7A9ftd0FyYg8i35Ftbj94xGQ6P0AZ

In order to provide as timely a service as possible, we ask that your revision is resubmitted within three months after receipt of this request. If an extension is needed, please send a request, along with a brief explanation, to the editorial office at plantdirect@wiley.com .

Thank you very much for giving us an opportunity to review your work. I look forward to receiving the next version.

Sincerely,
Marna Yandeau-Nelson

--- Editor comments:

After careful review, we feel that the submitted manuscript does not fully meet Plant Direct's publication criteria. We invite you to submit a revised manuscript that addresses the points outlined by the reviewers and the academic editor.

These points include:
1. Provide appropriate details in the methods and figure legends as appropriate regarding both biological and technical replication (See Reviewer 1 comments).
2. Expand the Discussion to cover the potentially unexpected observation that NPQ decreases with increasing AI concentration at the first two time points (MT1 and MT2), but these parameters both decrease at increasing Al concentration at MT3 and MT4. rETR shows a similar change in Al concentration response - in this case, increasing at MT1 and MT2, and decreasing with increasing Al concentration in MT3 and MT4. Although these opposite trends for both traits were reported in the Results they are not discussed or put in context of what is reported in the literature. Mechanistic insights in the Discussion would be useful.
3. The authors conclude that these above-ground Al-induced phenotypes identified by high-throughput imaging phenotyping could be used "in remote sensing studies to map Al-toxic soils". However, many of the measured phenotypes are likely to result from other stresses as well. It seems that additional work would be needed to identify Al-specific parameters, and some discussion on this may be needed.

Additional Minor Edits:
Line 194: HDS should be HSD (Honestly Significant Difference)
Line 248: Cite Table S2B for Rred, Rgreen, etc.
Line 268: provide a conceptual definition of "digital volume", as this is not a commonly reported parameter.
Supplemental Tables 1 and 2: State in the legend or in table what values are being presented, presumably p-values.

-- Reviewer comments:
Reviewer #1:

Szőke et al. conducted a comprehensive analysis using image-analysis phenotyping to evaluate the responses of corn seedlings to different Al toxicity conditions. While the study provides valuable descriptive results based on a single genotype, it lacks mechanistic insights. Furthermore, several areas require clarification and correction:
Comments:
(1) The manuscript does not specify the number of replicates used in the different analyses. Please provide this information to ensure the robustness and reproducibility of the results.
(2) In Figure 1, the false color scale is missing numerical indicators for high and low values. Adding these numbers would enhance the interpretability of the data.
(3) NPQ Value Discrepancies: There is an inconsistency in the non-photochemical quenching (NPQ) values under different Al concentrations. Specifically, higher Al3+concentrations decrease NPQ in MT1 and MT2 but increase NPQ in MT3 and MT4. An explanation for these contrasting responses should be provided to better understand the underlying physiological mechanisms.
(4) There are errors in the units presented in the manuscript:
Lines 136 and 141: The light unit should be written as "µmol m−2 s-1" instead of "µM m−2 s-1".
(5) In Figure 1, the treatment notation should be corrected to "400 µM Al" rather than "400 µM Al L-1".
(6) The title of Table 1 should be placed above the table for consistency with standard formatting practices.

Reviewer #2:

The authors tried to design, perform, and present valuable research about "Quantifying Aluminium Toxicity Effects on Corn Phenotype Using Advanced Imaging Technologies". I have some comments and suggestions for the current manuscript. After carefully reading the manuscript, I recommend acceptance.
Minor comments:
L150 Please move the title of table 1 to the top of the table.
Please check again the scientific names of plants and other cases in the reference section.

Decision Letter Round 2:

Plant Direct

Open Access

June 25, 2024

Dr. Boris Lazarevic
University of Zagreb Faculty of Agriculture
Department of Plant Nutrrition
Svetosimunska cesta 25
Zagreb, N/A
Croatia

MSID: 2024-01479R1
MS TITLE: Quantifying Aluminium Toxicity Effects on Corn Phenotype Using Advanced Imaging Technologies

Dear Dr. Boris Lazarevic:

I am pleased to inform you that your manuscript "Quantifying Aluminium Toxicity Effects on Corn Phenotype Using Advanced Imaging Technologies" has been accepted for publication in Plant Direct.

Your article will appear online in the next available issue of Plant Direct. To ensure your article gets published as quickly as possible, please pay attention to the steps detailed below. We have found that most of the delays happen at this stage, especially at the payment stage, so please respond as quickly as possible when prompted.

License Agreement: Once your article has been accepted it will move to Production and undergo admin and file checking - you may receive an email with any queries we have at this stage. When all required items are received by the publisher and queries resolved, the corresponding author will receive an email from Wiley's Author Services system which will ask them to log on at https://authorservices.wiley.com/bauthor and will present them with the appropriate license for completion. Your article cannot be published until both the signed license agreement and payment of the article fee have been received.

Payment of the Open Access Article Publication Fee: All articles published in Plant Direct are fully open access: immediately and freely available to read, download and share. Plant Direct charges a publication fee to cover the publication costs. The corresponding author for this manuscript should have already received a quote from the payments team (cs-openaccess@wiley.com) with the estimated article publication fee; please email cs-openaccess@wiley.com if this has not been received. The corresponding author should log on

to the Wiley Author Services site, where the publication fee can be paid by credit card or an invoice. Pro Forma can also be requested. Payment of the publication charge must be received before the article will be published online.

Proofs: You will have the opportunity to look over your paper once more when you receive the author proofs for your article. The proofs will be with you in approximately two weeks. Please note that, in addition to publishing reviewer comments, the author's responses to review comments will also be published alongside the final version of the paper. If you would not like the author's responses to be published, please contact the editorial office at plantdirect@wiley.com .

Promotion of your article: You can help your research get the attention it deserves! Check out Wiley's free Promotion Guide for best-practice recommendations for promoting your work at www.wileyauthors.com/eeo/guide. And learn more about Wiley Editing Services which offers professional video, design, and writing services to create shareable video abstracts, infographics, conference posters, lay summaries, and research news stories for your research at www.wileyauthors.com/eeo/promotion.

Thank you again for your contribution to Plant Direct. If you have any questions, feel free to contact the editorial office at plantdirect@wiley.com .

Sincerely,

Marna Yandeau-Nelson

Gustavo MacIntosh

Editor, Plant Direct

-- Editor comments
We thank the authors for submitting a revised manuscript that addressed the reviewer and academic editor's comments and concerns. This manuscript meets Plant Direct's publication criteria, and we are pleased to publish this work.

-- Reviewer comments:

Author's response to reviewer:

Dear Editor,

Thank you for allowing us to submit a revised draft of the manuscript "Quantifying Aluminium Toxicity Effects on Corn Phenotype Using Advanced Imaging Technologies" for publication in Plant Direct. We appreciate the time and effort you and the reviewers dedicated to providing feedback on our manuscript and are grateful for the insightful comments and valuable improvements to our paper. We have incorporated all of the suggestions you and the reviewers made. Those changes are highlighted within the revised manuscript file with tracked changes Please see below, in blue, for a point-by-point response to the reviewers' comments and suggestions. All line numbers refer to the revised manuscript file with tracked changes.

Comments from Academic Editor:

1. Provide appropriate details in the methods and figure legends, as appropriate, regarding both biological and technical replication (See Reviewer 1 comments).

Author response:

Please see Lines 126-127. We have added the sentence: Ten uniformly developed plants were grown in each treatment and used as replicates.

The number of plants has also been included in the figure captions: "(ten plants per treatment)". Please see the Lines 236 (Figure 2), 260(Figure 3) and 277 (Figure 4).

2. Expand the Discussion to cover the potentially unexpected observation that NPQ decreases with increasing AI concentration at the first two time points (MT1 and MT2), but these parameters both decrease at increasing Al concentration at MT3 and MT4. rETR shows a similar change in Al concentration response - in this case, increasing at MT1 and MT2, and decreasing with increasing Al concentration in MT3 and MT4. Although these opposite trends for both traits were reported in the Results they are not discussed or put in context of what is reported in the literature. See Reviewer 1 comments.

Author response:

The discussion about the NPQ and rETR has been added. Please see the Line number 366-382. Due to this new paragraph in the discussion, several new references are used and included in the list of references. Gong et al., 2020 (Line number 505); Chen and Gallie, 2008 (Line number 476); Grace and Logan,1996 (Line number 510), and Nicol et al., 2019 (Line number 577).

"During the different measurements (from MT1 to MT4), chlorophyll fluorescence parameters NPQ, qN, ϕnpq, and rETR showed inconsistent behaviour. Namely, higher Al3+ concentrations decreased NPQ, qN, ϕnpq, and increased rETR in MT1 and the opposite, increased NPQ, qN, and ϕnpq, and decreased rETR in MT3 and MT4. Similarly, the exposure to cadmium toxicity caused a decrease in NPQ, the quantum of regulated energy dissipation, and the quantum yield of non-regulated energy dissipation in two Buddleja species (Gong et al., 2020). These results could be explained by increased antioxidative mechanisms, such as the Mehler-peroxidase pathway (Grace and Logan,1996) during the early stage of the stress. This decrease in non-photochemical quenching parameters and related electron consumption can be attributed to a reduction in the induction of NPQ, as well as a shift in the balance between the quickly and slowly reversible components of NPQ (Chen and Gallie, 2008). However, the specific sites and mechanisms of NPQ remain elusive (Nicol et al., 2019). In addition, the prolonged exposure to the Al toxicity caused an increase in NPQ and a decrease in rETR, indicating the exhaustion of these protective mechanisms. These findings suggest a complex interplay between antioxidative mechanisms and NPQ in plants, with implications for plant acclimation to Al stress."

3. The authors conclude that these above-ground Al-induced phenotypes identified by high-throughput imaging phenotyping could be used "in remote sensing studies to map Al-toxic soils". However, many of the measured phenotypes are likely to result from other stresses as well. It seems that additional work would be needed to identify Al-specific parameters, and some discussion on this may be needed.

Author response:

The paragraph which discusses the possible effect of other stresses causing similar phenotypic symptoms to those found under the Al toxicity has been added. Please see Line numbers 394-401.

"Our study indicates that Al toxicity in corn can be assessed very early (after four days of exposure) using non-destructive image-based technology. However, Al toxicity in plants can cause a variety of disorders, among others: root growth inhibition, nutrient uptake and transport disruption and oxidative stress (Bojórquez-Quintal et al., 2017; Kochian et al., 2015; Lidon and Barreiro, 2002; Zhang et al., 2019). Therefore, the phenotypic symptoms found under conditions of Al toxicity may be caused by complex interactions between the primarily toxic effect of aluminium and other physiological disorders that occur under conditions of acidic soil and aluminium toxicity."

Due to this new paragraph in the discussion, two new references were used and included in the list of references. Please see Bojórquez-Quintal et al., 2017 (Line number 465) and Zhang et al., 2019 (Line number 636).

Also, the conclusions are slightly changed. Please see the Line numbers 408-414

Although this study was performed under controlled conditions, and the results should be confirmed under field conditions with combinations of different stresses, such as nutrient deficiencies and oxidative stress, it demonstrated the possibility of using selected phenotypic traits in remote sensing studies to map Al-toxic soils and/or in high-throughput phenotyping studies to screen Al-tolerant genotypes.

4. Line 194: HDS should be HSD (Honestly Significant Difference)

Author response:

Acknowledged. We have corrected this.

Please see the Line number 197

5. Line 248: Cite Table S2B for Rred, Rgreen, etc.

Author response:

Acknowledged. The citation for the supplementary table S2B is now included in this sentence. Please see the Line number: 251

6. Line 268: provide a conceptual definition of "digital volume", as this is not a commonly reported parameter.

Author response:

Acknowledged. The equation for the digital volume is included in the figure captions. Please see figure 4. caption. Line number: 277

7. Supplemental Tables 1 and 2: State in the legend or in table what values are being presented, presumably p-values.

Author response:

Acknowledged. The values represent p-values. This is now indicated in the caption of the Supplementary tables.

Reviewer #1 comments:

1. The manuscript does not specify the number of replicates used in the different analyses. Please provide this information to ensure the robustness and reproducibility of the results.

Author response:

Acknowledged. Please see Lines 126-127. We have added the sentence: Ten uniformly developed plants were grown in each treatment and used as replicates.

The number of plants has also been included in the figure captions: “(ten plants per treatment)”. Please see the Lines 236 (Figure 2), 260(Figure 3) and 277 (Figure 4).

In Figure 1, the false color scale is missing numerical indicators for high and low values. Adding these numbers would enhance the interpretability of the data.

Acknowledged. Please see figure 1. The numerical indicators are added below each false color scale.

2. NPQ Value Discrepancies: There is an inconsistency in the non-photochemical quenching (NPQ) values under different Al concentrations. Specifically, higher Al3+concentrations decrease NPQ in MT1 and MT2 but increase NPQ in MT3 and MT4. An explanation for these contrasting responses should be provided to better understand the underlying physiological mechanisms.

Author response:

The discussion about the NPQ and rETR has been added. Please see the Line number 360-376.

Due to this new paragraph in the discussion, several new references are used and included in the list of references. Please see Gong et al., 2020 (Line number 505); Chen and Gallie, 2008 (Line number 476); Grace and Logan,1996 (Line number 510), and Nicol et al., 2019 (Line number 577).

"During the different measurements (from MT1 to MT4), chlorophyll fluorescence parameters NPQ, qN, ϕnpq, and rETR showed inconsistent behaviour. Namely, higher Al^{3+} concentrations decreased NPQ, qN, ϕnpq, and increased rETR in MT1 and the opposite, increased NPQ, qN, and ϕnpq, and decreased rETR in MT3 and MT4. Similarly, the exposure to cadmium toxicity caused a decrease in NPQ, the quantum of regulated energy dissipation, and the quantum yield of non-regulated energy dissipation in two Buddleja species (Gong et al., 2020). These results could be explained by increased antioxidative mechanisms, such as the Mehler-peroxidase pathway (Grace and Logan,1996) during the early stage of the stress. This decrease in non-photochemical quenching parameters and related electron consumption can be attributed to a reduction in the induction of NPQ, as well as a shift in the balance between the quickly and slowly reversible components of NPQ (Chen and Gallie, 2008). However, the specific sites and mechanisms of NPQ remain elusive (Nicol et al., 2019). In addition, the prolonged exposure to the Al toxicity caused an increase in NPQ and a decrease in rETR, indicating the exhaustion of these protective mechanisms. These findings suggest a complex interplay between antioxidative mechanisms and NPQ in plants, with implications for plant acclimation to Al stress."

3. There are errors in the units presented in the manuscript:

Author response

Acknowledged.

Please see Lines 138, 142, 145, and 157. The light units are corrected, now they are written as "µmol m−2 s-1" instead of "µM m−2 s-1".

4. In Figure 1, the treatment notation should be corrected to "400 µM Al" rather than "400 µM Al L-1".

Author response.

Acknowledged. We have corrected this; please see Figure 1.

5. The title of Table 1 should be placed above the table for consistency with standard formatting practices.

Author response

Acknowledged. The title of Table 1 is placed above the table. Please see Line 152.

Reviewer #2 comments:

1. L150 Please move the title of table 1 to the top of the table.

Author response

Acknowledged. The title of Table 1 is placed above the table. Please see Line 152.

2. Please check again the scientific names of plants and other cases in the reference section.

Author response

Acknowledged. We have corrected the scientific names of plants in the reference section.

Please see lines: 464, 538, 600
